# A randomized controlled trial for overweight and obesity in preschoolers: the More and Less Europe study - an intervention within the STOP project

**DOI:** 10.1186/s12889-019-7161-y

**Published:** 2019-07-15

**Authors:** Anna Ek, Christine Delisle Nyström, Adela Chirita-Emandi, Josep A. Tur, Karin Nordin, Cristina Bouzas, Emma Argelich, J. Alfredo Martínez, Gary Frost, Isabel Garcia-Perez, Marc Saez, Corina Paul, Marie Löf, Paulina Nowicka

**Affiliations:** 1 Division of Pediatrics, Department of Clinical Science Intervention and Technology, Karolinska Institutet, Stockholm, Sweden; 2 Department of Biosciences and Nutrition, Karolinska Institutet, Stockholm, Sweden; 3 Genetics Department, University of Medicine and Pharmacy “Victor Babes”, Timisoara, Romania; 4 “Louis Turcanu” Clinical Emergency Hospital for Children, Timisoara, Romania; 5 Research Group on Community Nutrition & Oxidative Stress, University of the Balearic Islands, Palma de Mallorca, Spain; 6 CIBER of Physiology of Obesity and Nutrition (CIBEROBN), Instituto Carlos III, Madrid, Spain; 7 Department of Nutrition, Food Science, and Physiology, Centre for Nutrition Research, University of Navarra, Pamplona, Spain; 8 IMDEA Food Precision Nutrition, Madrid, Spain; 9 Section for Nutrition Research, Department of Medicine, Imperial College London, Hammersmith Campus, London, UK; 10 Division of Systems and Digestive Medicine, Department of Surgery and Cancer, Faculty of Medicine, Imperial College London, South Kensington Campus, London, UK; 11 Research Group on Statistics, Econometrics and Health (GRECS), University of Girona, Campus de Montilivi, Girona, Spain; 12 CIBER of Epidemiology and Public Health (CIBERESP), Instituto Carlos III, Madrid, Spain; 13 Pediatrics Department, University of Medicine and Pharmacy “Victor Babes”, Timisoara, Romania; 14 2nd Pediatrics Clinic, Clinical Emergency County Hospital Timisoara, Timisoara, Romania; 15 Department of Medical and Health Sciences, Linköping University, Linköping, Sweden; 16 Department of Food Studies, Nutrition, and Dietetics, Uppsala University, Uppsala, Sweden

**Keywords:** Children, Family, mHealth, Obesity, Overweight, Treatment, Stop

## Abstract

**Background:**

Childhood overweight and obesity is a serious public health issue with an increase being observed in preschool-aged children. Treating childhood obesity is difficult and few countries use standardized treatments. Therefore, there is a need to find effective approaches that are feasible for both health care providers and families. Thus, the overall aim of this study is to assess the acceptance and effectiveness of a parent support program (the More and Less, ML) for the management of overweight and obesity followed by a mobile health (mHealth) program (the MINISTOP application) in a socially diverse population of families.

**Methods/design:**

A two-arm, parallel design randomized controlled trial in 300 2-to 6-year-old children with overweight and obesity from Romania, Spain and Sweden (*n* = 100 from each). Following baseline assessments children are randomized into the intervention or control group in a 1:1 ratio. The intervention, the ML program, consists of 10-weekly group sessions which focus on evidence-based parenting practices, followed by the previously validated MINISTOP application for 6-months to support healthy eating and physical activity behaviors. The primary outcome is change in body mass index (BMI) z-score after 9-months and secondary outcomes include: waist circumference, eating behavior (Child Eating Behavior Questionnaire), parenting behavior (Comprehensive Feeding Practices Questionnaire), physical activity (ActiGraph wGT3x-BT), dietary patterns (based on metabolic markers from urine and 24 h dietary recalls), epigenetic and gut hormones (fasting blood samples), and the overall acceptance of the overweight and obesity management in young children (semi-structured interviews). Outcomes are measured at baseline and after: 10-weeks (only BMI z-score, waist circumference), 9-months (all outcomes), 15- and 21-months (all outcomes except physical activity, dietary patterns, epigenetics and gut hormones) post-baseline.

**Discussion:**

This study will evaluate a parent support program for weight management in young children in three European countries. To boost the effect of the ML program the families will be supported by an app for 6-months. If the program is found to be effective, it has the potential to be implemented into routine care to reduce overweight and obesity in young children and the app could prove to be a viable option for sustained effects of the care provided.

**Trial registration:**

ClinicalTrials.gov NCT03800823; 11 Jan 2019.

## Background

According to the World Health Organization childhood obesity is one of the gravest public health challenges of today’s society [1], with approximately 108 million 2- to 19-year-old children being classified as having obesity [2]. More specifically, in children less than 5 years, there has been a swift increase in childhood overweight and obesity and if these trends continue it is predicted that 70 million children will be overweight or obese by 2025 [3]. These statistics are concerning as Geserick et al. [4] found that 90% of 3 year olds with obesity still had overweight or obesity in adolescence. Furthermore, for those adolescents with overweight or obesity, the majority of weight gain happened between two and 6 years of age [4]. Thus, this demonstrates the need for evidence-based treatment programs in the pre-school years in order to attempt to rectify the increased prevalence of childhood overweight and obesity.

According to Colquitt et al. [5] for children under 6 years of age multicomponent interventions (i.e., diet, physical activity, and behavioral interventions) seem to be effective at treating overweight and obesity. However, the authors did state that evidence is limited [5]. To date, the majority of the treatment interventions for overweight and obesity use face-to-face delivery methods [6]. A recent meta-analysis by Ling et al. [6] found small effect sizes on treatment interventions for preschool-aged children for body mass index (BMI) (− 0.28 kg/m^2^, *p* < 0.001) using various in person delivery methods. Furthermore, the More and Less (ML) study found that at the 12-month follow-up, a 10-week group treatment program focusing on parenting practices had a greater reduction in BMI z-scores than standard treatment in health care (− 0.30 vs. -0.07, *p* < 0.05). An even greater reduction was observed in the intervention group who received booster sessions (a 30-min phone call every 4 to 6 weeks over a 9-month period) [7]. These results are promising; however, sustained contacts with families after treatment programs are burdensome on both health care providers and participants, which makes it difficult to scale-up. Therefore, different types of boosters need to be used in order to reduce the burden on both health care and participants.

The universal use of smartphones makes the use of mobile health (mHealth) an option for boosting the effects of treatment programs. mHealth is increasingly being used for promoting healthy habits and as treatment of many types of health conditions and diseases. In adults, two meta-analyses have found that mHealth interventions focusing on weight loss significantly decreased participants’ weight in the intervention groups compared to the control groups [8, 9]. In children and adolescents few studies have utilized mHealth in the prevention or treatment of obesity [10–14] and hardly any have been conducted in the preschool-age group [15, 16]. The Mobile-based Intervention Intended to Stop Obesity in Preschoolers (MINISTOP) trial was a mHealth obesity prevention intervention that was developed and led by Marie Löf and her team to improve 4-year-old children’s body composition, dietary, physical activity, and sedentary behaviors [17, 18]. The MINISTOP intervention had a significant effect on a composite score composed of body composition, diet, and physical activity variables, with this effect being more evident among children with a higher fat mass index [18]. There are numerous advantages of mHealth over conventional intervention approaches such as: the programs can be delivered any time and place; are interactive; can be tailored to different groups (e.g., translated into multiple languages); and reduces burden on health care professionals and participants. These advantages further motivates the use of mHealth in families with young children with overweight and obesity.

The mechanisms that drive weight gain such as epigenetics and gut hormones are still unclear [19, 20]. Epigenetics has received attention during the recent years for the putative involvement in transmitting obesity risk to offspring and in the heritable regulation of gene expression without altering their coding sequence [21]. The most relevant epigenetic mechanisms involved in gene activity control are histone modifications, non-coding RNAs (ncRNA) and DNA methylation [20]. Further, obesity has been associated with the epigenetic modulation of several genes. For example, a relationship has been reported between increased BMI and adiposity as well as higher DNA methylation levels at the hypoxia-inducible transcription factor 3A (HIF3A) gene [22]. Moreover, an increased methylation in the gene RXRA measured at birth has been associated with greater adiposity in later childhood [23]. Two other investigations identified a strong correlation between obesity and serum levels of micro RNA (miR)-122 and miR-519d [24] and found DNA methylation to be related to insulin resistance [25]. However, these findings need to be confirmed and further explored in young children.

Another field of interest for obesity is the gastrointestinal tract (GIT) [26]. The GIT plays an important role in acute appetite regulation through a number of mechanisms: (1) the release of hormones that play a role in appetite regulation such as anorectic hormones (Peptide YY, PYY, and glucagon-like peptide, GLP-1) and orexogenic gut hormones (e.g., ghrelin), (2) the enteric nervous system and signals through the vagus to the brain to influence appetite and (3) secondary to stimulating signals from other organs such as liver adipose. Previous research in adults has demonstrated that the infusion of the GIT anorectic hormones PYY and GLP-1 at physiological doses has profound effects to suppress appetite [26]. Also weight loss appears to lead to a suppression of PYY and GLP-1 suggesting a role in the feelings of hunger during weight reduction. However, evidence of the role of GIT hormones in overweight and obesity among young children is sparse.

A major challenge in the management of obesity in both adults and children is understanding what people eat. Most dietary assessment methodologies use methods of self-reported food intake which is a subject to large misreporting error [27, 28]. It is therefore impossible to understand what children eat. Garcia et al. has developed a new metabolomic methodology of dietary assessment using urine, which is not subject to the same misreporting errors [29]. This method has been validated in adults. Our aim is to do this is children.

To the best of our knowledge there is no study to date that has the ambition to assess a broad array of key biological and social determinants of obesity in young children. This study protocol outlines the design of a multi-country study that incorporates both a parent support program and mHealth in an overweight and obesity intervention in 2- to 6-year-old children with overweight and obesity.

### Aim

The overall aim of this study is to assess the feasibility, acceptance and effectiveness of an overweight and obesity intervention in a socially diverse population of families. The specific aims are:

1. To determine the effectiveness on child weight status (BMI z-score) of a 10-week parent support program delivered in groups focusing on evidence-based parenting practices (the ML program) followed by a mHealth component for 6-months (the MINISTOP application, app) for overweight and obesity in preschool-aged children.
2. To assess change in secondary outcomes, which are: waist circumference, child eating behavior, parental feeding practices, and physical activity.
3. To assess epigenetic mechanisms and physio pathological processes underlying childhood obesity including the role of gut hormones.
4. To assess and validate child food intake with metabolic markers in urine metabolomics.
5. To evaluate the feasibility of recruitment (facilitators and barriers), attrition and acceptability of the ML program, the standard treatment and the overall acceptance of overweight and obesity management according to patients and care providers.

### Hypotheses

Our central hypothesis is that the intervention (the ML program followed by the MINISTOP app for boosting) will be more effective in decreasing children’s BMI z-score (primary outcome), improving eating and feeding behaviors, and physical activity (secondary outcomes) compared to standard care. Another study hypothesis is that the intervention will produce changes in urinary metabolites, which will serve as biomarkers of the nutritional outcomes or as targets for application. We also hypothesize that the parent program and the mHealth intervention will be well accepted by families and caregivers.

## Methods

### Study design

**Fig. 1:**
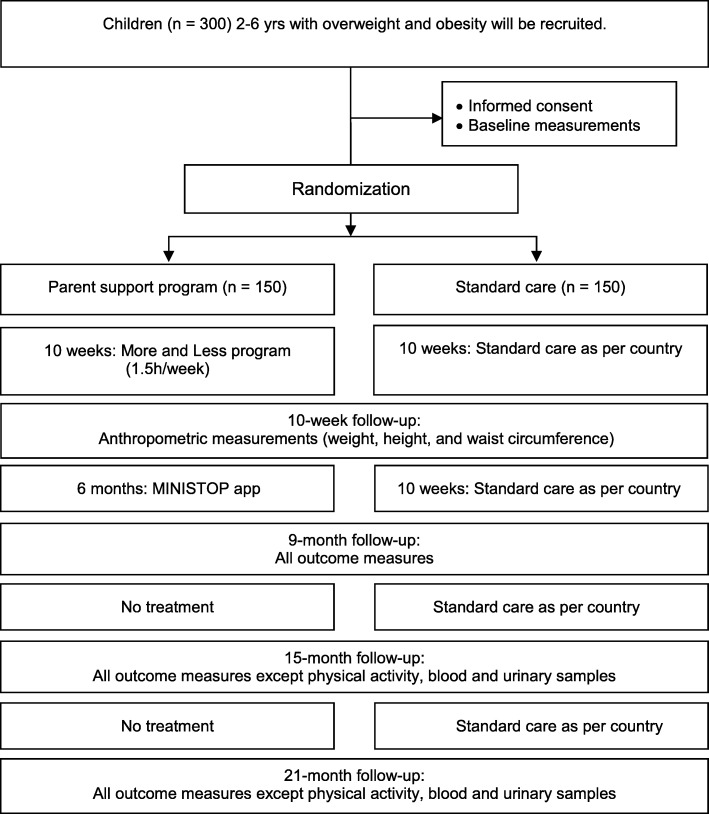
Flow-chart of the More and Less Europe trial design

ML Europe is a two-arm parallel design randomized controlled trial (RCT) comparing overweight and obesity treatments in 2-to 6-year-old children in three countries (Romania, Spain, and Sweden). Following baseline assessments, participants will be randomized into the intervention and control group in a 1:1 ratio. The intervention group receives a 10-week parent support program (the ML program) which focuses on evidence-based parenting practices [7, 30] followed by a previously validated 6-month mHealth program (the MINISTOP app, PI: M Löf) to support healthy lifestyle changes [17, 18]. The control group receives standard treatment as offered in the country of participation. The different interventions are described in greater detail below. Assessments will be conducted at 10 weeks, 9 months, 15 months, and 21 months post-baseline (see Fig. 1 for study outline). This study protocol follows the SPIRIT 2013 statement [31, 32].

### Sample size and power calculation

Based on power calculations, 75 children are needed in each group (adjusted for drop-out) to detect a difference of 0.3 BMI z-score with 85% power at the 9-month follow-up between the intervention and control group. These calculations are based on a previous study in this age group [33]. Thus, each site aims to recruit 100 participants to ensure adequate power.

### Participants, eligibility, and recruitment

In total, we aim to include 300 families (*n* = 100 in Romania, Spain, and Sweden, respectively). To be included in this study: children must be between 2 and 6 years old and have overweight or obesity as classified by international cut-offs [34]; have no other underlying medical condition(s); the child has not started any treatment for overweight or obesity; and at least one parent has to have the ability to communicate in Romanian, Spanish, or Swedish depending on the country of participation. Parents who do not own a smartphone compatible with the MINISTOP app will be excluded from this study (i.e., version 10.0 or higher for iOS or version 5.0 or higher for Android).

Recruitment will follow a standardized protocol for all countries. In Romania, family physicians and pediatricians will be involved to hand out information regarding the study to families with 2- to 6-year-olds with overweight or obesity. Parents who want to learn more about the study are provided with a phone number, email address, web page and Facebook page with information of how to contact the research group. Participants will also be recruited, as self-referrals, using an official page for the study on Facebook to be shared with specialized groups.

In Spain, families with children who attend weight and height assessments at their pediatricians at primary care health centers and hospitals will be asked to participate in the study. If the parents are interested in participating, the pediatrician will schedule a visit within a maximum of 7 days to provide them with more detailed information regarding the study and for them to sign the informed consent.

Finally, in Sweden, the recruitment methods have been previously described in detail [7, 30]. Briefly, recruitment is done primarily at primary child health care centers, where all parents of children from birth to 5 years of age are offered free, yearly check-ups. If overweight or obesity is detected the nurse provides a verbal and a short one-page explanation of the study. If the parent(s) are interested in participating the nurse sends a referral to the research group that will send out more detailed information regarding the study together with a consent letter. After 1 week, a member from the research team will contact the families to answer any questions that they have. Recruitment is also conducted at secondary health care (i.e., out-patient pediatric clinics). Additionally, self-recruitment is being done through newspaper ads as well as by placing posters on primary health care bulletin boards.

For all countries, after fully informing the families, if they still want to participate they send back the signed consent letter, which is subsequently signed by a member of the research team and a copy is sent back to the family. A time for baseline assessments is then scheduled with the research group.

### Randomization and blinding

After the consent form has been signed, the participants are randomly allocated to either the intervention group (parent support program and mHealth booster) or the control group (standard care as per country) at a 1:1 ratio via a random allocation sequence list (in blocks of three). The sequence list was generated using free software environment for statistical computing and graphics R (version 3.5.1) [35]. The random allocation sequence is managed by a person who has no relationship with recruitment or treatment and opaque envelopes are used to ensure concealment. Those assessing the outcomes are blinded to the treatment allocation; however, owing to the nature of the intervention participants are not blind to their allocation.

### Intervention

#### The More and Less program

The ML program is based on the Keeping Foster and Kin Parents Supported and Trained (KEEP) parenting program, which has been tested in multiple settings [36–39]. KEEP is based on Bandura’s Social Learning Theory [40] and Patterson’s Social Interaction Learning Theory [41, 42]. The key concept of the programs is to support parents in evidence-based parenting practices, especially regarding positive reinforcement and limit setting, in order to improve parent and child communication. In ML, the improved communication lays the foundation for parents to support a healthy lifestyle for the child.

The ML program is comprised of 10 weekly sessions (1.5 h/week) and is culturally adapted for Romanian, Spanish, and Swedish families with preschool aged children with overweight or obesity. Table 1 displays the content of the ML program [7, 30]. Beyond the evidence-based parenting practices, the program includes content regarding healthy food habits, physical activity habits, as well as techniques to help parents regulate emotional control. Each session begins with a theoretical introduction to a parenting skill, the focus of the session is then discussed and practice is done through role play and homework assignments. To facilitate the implementation of the ML program it follows a manual where the sessions are described with precise instructions to the group leaders (2 per group). The parents receive a manual which summarizes what has been discussed during each session. For parents who are unable to attend sessions, the parental manual is sent home to the family and the family is contacted by phone for a brief review of the session. To facilitate session attendance the time and location for the groups are planned to suit the parents. Child care is also provided during the sessions.

**Table 1:** Session content of the More and Less program and themes included in the MINISTOP app

*More and Less Parenting Program*	
Session	Content
1	Welcome and overview
2	Food and play: When more? When less?
3	Parents as teachers: cooperation and energy balance
4	Parents as teachers: to teach children new behaviors
5	Rewards and incentives
6	Pre-teaching
7	Parents as teachers: limit setting strategies
8	Power struggles: to avoid and to handle them
9	More support – Less stress
10	Summary: parenting, food and play – to prepare for the future
*MINISTOP app*	
Theme	Content
1	Healthy foods in general
2	Breakfast
3	Healthy small meals
4	Physical activity and sedentary behavior
5	Candy and sweets
6	Fruits and vegetables
7	Drinks
8	Eating between meals
9	Fast food
10	Sleep
11	Foods outside the home
12	Foods at special occasions

The ML group leaders received an initial 4 day training in child overweight and obesity management and in the ML program content. The training was provided by the ML program developers PN and AE. During the training the sessions of the program were thoroughly discussed and the group leaders were trained in how to deliver the program by acting as group leaders while the other participants acted as parents. The training of group leaders will continue by external supervision after each weekly session for the first group in all countries. The group leaders will be asked to watch the filmed sessions and reflect on how they delivered the program. In Sweden and in Spain, groups will be held in health care facilities and in Romania in university facilities.

#### The MINISTOP app

The MINISTOP app was developed and evaluated in a population based study with preschool aged children (PI: Marie Löf) and has been previously described in detail [17, 18]. Briefly, MINISTOP comprises of an extensive program of information and push notifications built using current guidelines for a healthy diet and physical activity in pre-school aged children [43]. Over the 6-month period 12 themes will be covered (Table 1). A new theme is introduced bi-weekly, with parents being alerted by a push notification when this happens. Every theme is split into three parts (general information; advice; and strategies to change unwanted behavior). Through the app, parents have the ability to register their child’s consumption of sugar sweetened beverages, candy, fruits and vegetables, and physical activity and sedentary behavior. Parents then receive feedback on the registered parameters at the end of every week. Reminder messages are sent out to parents if they have not been in the app after a couple of days [17].

Two days before the tenth and final session of the ML program, parents receive an email with a username and password for the MINISTOP app as well as a text message with a link to download the app. At the final session, the ML program leaders will ensure that all parents were able to download the app and sign in. Thereafter, they will explain how the app works to the parents and answer any questions that they may have.

### Control

The weight management offered to the control group follows the standard care procedure for each country of participation. In Romania and Spain, the control group receives an evaluation of a one-day food frequency questionnaire as well as a 30-min consultation with a doctor that is a specialist in childhood nutrition, where healthy lifestyle recommendations are made. The parents also receive a hand-out which provides general recommendations for healthy food and physical activity in 2 to 6 year olds. Furthermore, in Romania the children are re-evaluated after 3 months during a 15-min consultation. In Sweden, the control group receives standard care according to the Action plan for overweight and obesity for Stockholm County [44]. Children with overweight and children with obesity younger than 4 years receive support from their child health care nurse. Children older than for 4 years with obesity are followed in an outpatient pediatric clinic with yearly visits to a pediatrician and follow-up visits to a pediatric nurse, approximately 5 visits (30 min in duration) per year [7]. The treatment centers around supporting the family in creating healthy diet and physical activity habits for the child. Children may also be referred to dieticians, psychologists or physiotherapists.

### Measures

Outcome measures are collected at baseline, 10 weeks, 9 months, 15 months, and 21 months post baseline. Table 2 presents when outcome measures are assessed and the instruments used to assess child and parental behaviors are displayed in Table 3.

**Table 2:** Socio-demographic characteristics and outcome measures collected at different time points

Outcomes	Measure	Baseline	10 weeks	9 months	15 months	21 months
Child						
Weight and height (BMI z-score)	Measured by health care professionals	x	x	x	x	x
Waist circumference		x	x	x	x	x
Date of birth	Child background questionnaire	x				
Country of birth		x				
Sex		x				
Health status		x		x	x	x
Family structure		x		x	x	x
Daycare		x		x	x	x
Visits to health care regarding weight		x		x	x	x
Screen time		x		x	x	x
Breakfast consumption		x		x	x	x
Sugar sweetened drinks consumption		x		x	x	x
Eating behavior	Child Eating Behavior Questionnaire	x		x	x	x
Physical activity / sedentary behavior	ActiGraph wGT3x-BT accelerometer	x		x		
Food intake	Urine samples, 24 h dietary recall	x		x		
Gut hormones	Fasting blood samples	x		x		
Epigenetic markers	Fasting blood samples	x		x		
Parent						
Weight and height (BMI)	Parent background questionnaire	x		x	x	x
Date of birth		x				
Country of birth		x				
Sex		x				
Education level		x				
Health status		x		x	x	x
Occupation status		x		x	x	x
Income		x		x	x	x
Social and economic support from network		x		x	x	x
Perceived level of comfortable life		x		x	x	x
Parenting behavior	Comprehensive Feeding Practices Questionnaire	x		x	x	x

Abbreviations: *BMI* body mass index

**Table 3:** Measures used in the study

Instrument, reference	Domains	No. items	Description
Child Eating Behavior Questionnaire (CEBQ),		35	
Wardle et al. 2001 [45]	*Food approach*		
	Food responsiveness	5	The child’s general appetite
	Enjoyment of food	4	The child’s interest in food
	Emotional overeating	4	If the child eats as a response to emotions
	Desire to drink	3	The child’s desire to drink
	*Food avoidance*		
	Satiety responsiveness	5	If the child gets full easily or not
	Slowness in eating	4	The child’s speed of eating
	Emotional undereating	4	If the child eats less in response to emotions
	Fussiness	6	The child eats a limited variety of food
Comprehensive Feeding Practices Questionnaire (CFPQ),		49	
Musher-Eizenman & Holub 2007 [46]	Monitoring	4	Parents keep track of child’s intake of less healthy foods
	Emotional regulation	3	Parents use food to regulate the child’s emotional stress
	Food as a reward	3	Parents use food as a reward for child behaviour
	Child control	5	Parents allow the child control of his/her eating behaviors and parent-child feeding interactions
	Modeling	4	Parents actively demonstrate healthy eating for the child
	Restriction for weight	8	Parents control the child’s food intake with the purpose of decreasing or maintaining the child’s weight
	Restriction for health	4	Parents control the child’s food intake with the purpose of limiting less healthy foods and sweets
	Teaching nutrition	3	Parents use explicit didactic techniques to encourage the consumption of healthy foods
	Encourage balance and variety	4	Parents promote well-balanced food intake, including the consumption of varied foods and healthy food choices
	Pressure to eat	4	Parents pressure the child to consume more food at meals
	Healthy environment	4	Parents make healthy foods available in the home
	Involvement	3	Parent’s encourage child’s involvement in meal planning and preparation

#### Primary outcome

BMI z-score is the primary outcome measure which is the most commonly used indicator of weight change in pediatric obesity studies [47]. The children’s weight and height will be measured to the nearest 0.1 kg and 0.1 cm, respectively. A fixed stadiometer is used to assess height and weight will be measured with the children wearing only underwear. BMI is derived as weight (kg) divided by height (m) squared. BMI z-scores are then calculated using age and gender specific reference values [34].

#### Secondary outcomes

##### Waist circumference

Waist circumference is measured at the mid-point between the lower rib and iliac crest to the nearest 0.1 cm using a non-elastic tape measurer.

Weight, height and waist circumference are measured three times and mean values are then calculated. All children are measured in a standardized manner by trained health care professionals using calibrated instruments.

##### Eating behavior

The children’s eating behavior is assessed using the Child Eating Behavior Questionnaire (CEBQ) [45]. It includes 35 items on eating styles comprising eight factors related to the risk of obesity. Parents rate each behavior on a five-point Likert scale (`never´, `rarely´, `sometimes´, `mostly´, and `always´ for items 1 to 13 and `disagree´, `slightly disagree´, `neutral´, `slightly agree´, and `agree´ for items 14 to 49). Mean scores for each sub-scale are calculated. This questionnaire has been found to have high internal reliability and good validity [45, 48–53].

##### Parenting behavior

The Comprehensive Feeding Practices Questionnaire (CFPQ) is used to measure parenting behavior [46]. The CFPQ is a parent-report instrument, designed to measure feeding practices of parents of children aged 2–8 years. It contains 49 items comprising 12 factors, where parents rate each behavior on a five-point Likert scale (`never´, `rarely´, `sometimes´, `mostly´, and `always´). The CFPQ has previously been validated in Brazilian preschoolers [54].

##### Physical activity and sedentary behavior

The ActiGraph wGT3x-BT accelerometer (ActiGraph Corp, Pensacola, USA, www.actiGraphcorp.com) is used to assess physical activity and sedentary behavior over seven consecutive 24 h periods. The ActiGraph will be attached the child’s non-dominant wrist and be worn at all times, except for water-based activities (e.g., showering/bathing or swimming). The recorded movements will be used to estimate time in various activity levels based on appropriate cut-points.

##### Metabolites of food intake

First void urinary samples will be collected from the children and will be used to assess metabolites of food intake. Two urine samples from the child will be collected by the parents at home six and 3 days before the visit to the research group. The third urine sample is collected on the morning of the visit to the research group. The urine metabolite analysis will be carried out as previously described [29]. In brief, urine samples will be measured by proton nuclear magnetic resonance (^1^H-NMR) spectroscopy. Global urinary ^1^H-NMR profiles will be used to predict the quality of the diet using the World Health Organization guidelines as a reference. Individual urinary metabolites associated with the intake of foods will be used to assess the dietary profile of the child. The Dietary Metabotype Score that embodies concentrations of urinary metabolites related to food components and adherence to diet will be developed and validated against one 24-h dietary recall with a parent. The 24-h recall will cover the day before the visit to the research group. For children attending preschool a food diary for teachers to fill out will be collected to cover the food intake not provided by the parent.

##### Epigenetic markers and gut hormones

Fasting blood samples are collected to assess reversibility of metabolic markers through epigenetic markers and the role of gut hormones.

Epigenetic markers

The epigenetic analysis is carried out in white blood cells, which require DNA extraction, bisulphite transformation, and analysis with Polymerase chain reactions (PCRs) or other technologies involving hypothesis driven methylation (CpGs). The unit of measurement/criteria is changes in percentage CpGs. The methodology has been explained in detail elsewhere [55, 56]. Methylation levels will be analyzed following standardized epigenetic methods after bisulphite conversion as described previously [55, 56] in hypothesis- driven specific CpGs.

Gut hormones

PYY concentrations will be measured using an in-house radioimmunoassay (RIA). The assays are highly sensitive and do not cross-react with other gut hormones. Separation of the antibody-antigen complexes from the free antigen is achieved by secondary antibody. The reported intra- and inter-assay variation is 5.8 and 9.8% respectively.

GLP-1 concentrations will be measured using an in-house RIA. This assay is highly specific and sensitive with the antibody cross reacting with 100% of all amidated forms of GLP-1. The assay does not cross react with glycine extended forms (GLP1–37 and GLP9–37) or any other gut hormones. The lowest level of GLP-1 that can be detected by this assay is 7.5 pmol/l. Separation of the antibody-antigen complexes from the free antigen is achieved by charcoal adsorption. The reported in-house intra- and inter-assay variation is 5.4 and 11.5% respectively.

##### Feasibility, attrition, and acceptability

Using semi-structured interviews, the facilitators and barriers of recruitment as well as attrition to the intervention (first 10 weeks, i.e., the ML parent program) and feasibility and acceptability of the MINISTOP app and of the standard care offered are assessed. Both parents and healthcare professionals are interviewed by trained research staff. During the interviews a set of questions are asked to all participants follow-up questions are however based on individual responses. The questions have been tested in pilot interviews with both parents and health care professionals. The interviews are recorded and fully transcribed. Interviews will be conducted before and after the intervention.

##### Sociodemographic data

At baseline parents are asked to fill out a background questionnaire for the child and themselves. Questions for the parent include: health status, sociodemographic factors and social support. For the child, questions include: country of birth, health status, family structure and lifestyle related questions such as food and screen time behaviors.

#### Adverse events

Adverse events will be monitored, reported and handled appropriately. The risks imposed by this research project are deemed to be low, i.e., the burden of the experiments for the research subjects is limited. It is important to note that blood samples collected in the study are optional and not a criteria for participation. However, blood samples are taken by experienced nurses and a pain reducing cream is used to reduce any discomfort. Urinary samples are none invasive and thus cause no risk to the participants. In addition, the investigators have extensive experience conducting behavioral weight control studies, and active efforts will be taken by the research staff to ensure the participating families’ safety. Other adverse events may include psychosocial burden that parents may experience when made aware about their child’s weight status and the sense of guilt that may arise. To handle that, already in the first session of the ML program causes and consequences are reviewed in a non-judgmental way. Also, potential impact on the child’s self-esteem and the way to talk about body weight and obesity with children, if necessary, are addressed.

### Data management

All collected data will be handled as approved by the ethical boards to protect confidentiality. Data is de-identified and entered manually into a database by research staff at the participating site where the data originated from. An identical database is used at each site. To ensure data quality and validity the researchers follow standard operation procedure protocols when entering data. The entered data will be double checked by the person entering the data and random checks will be performed regularly to ensure data validity. The database will be password protected and access is restricted to researchers with passwords. Original data forms will be stored in a secure place at each study site.

### Statistical analysis

Intention-to-treat analysis using generalized linear mixed models with repeated measures will be used to examine the effects of the intervention on primary (aim 1) and secondary outcomes (aim 2) for the total study population (i.e., all three sites). The link function for the primary outcome (BMI z-score) will be the identity and the Gaussian family (equivalent to a linear regression). In secondary outcomes we will use a Gaussian identity and family link function for waist circumference, physical activity and sedentary behavior, and a logarithmic link and Poisson family function (equivalent to a Poisson regression) for child eating behavior and parental feeding practices. A random effect for country will be used to account for the clustered study design. In the models, we will control for relevant covariates such as sex, age, parental weight status, education level, income and foreign background. Random intercept and a random slope for time will be included in the model to control those non-observed confounders specific to each child that could be constant or vary in time, respectively. Furthermore, interactions between variables will be estimated. If missing values in the outcomes (primary and secondary) are more than 10%, these will be imputed through a two-part model (also known as a model for semi continuous data). In this model, we would simultaneously estimate the probability of not being missing (first part) and the outcome (second part), using a mixed generalized linear model, in which we would include, as explanatory variables: age, sex, parental weight status, foreign background, educational level, and the random effects which are aforementioned.

Statistical tests and analyses of the interaction of phenotypical outcomes with epigenetics will include Manhattan plots, volcano plots, principal component analysis (PCA)/cluster, heatmaps, partial least square-discriminant analysis (PLS-DA), correlations and association studies, linear regression models, receiver operating characteristic (ROC) curves and these will be implemented as appropriate.

The means and medians for the gut hormone values before and after the intervention will be compared, using Student’s t test and Mann-Whitney U test, respectively. The differences will be adjusted in a generalized linear mixed model, with an identity link and Gaussian family, including the confounders, both observed and unobserved, indicated above.

For the validation of child food intake with metabolic markers in urine, the urinary dietary model will be derived using previously described methodology [29]. Comparison between the study groups will be carried out using PCA and Monte Carlo cross-validated partial least square-discriminant analysis (MCCV-PLS-DA) methodology. The relationship between dietary biomarkers and the dietary metabolite profile will be carried out using a generalized linear mixed model, with an identity link and Gaussian family, including, again, the confounders.

The semi-structured interviews with parents and health care professional will be fully transcribed verbatim and analyzed using thematic analysis [57].

For our analyses we will use R [35], STATA version 12.1 (StataCorp 2011, College Station, TX, USA) and SPSS Statistics (IBM, Armonk, NY, USA).

### Ethics approval

This trial was approved by: the Ethics Committee of Scientific Research in University of Medicine and Pharmacy “Victor Babes”, Timisoara, Romania, October 31st, 2018 (25/31.10.2018), the Balearic Islands Ethics Committee, Mallorca, Spain, February 13th, 2019 (IB 3814/18 PI), and the Research Ethics Committee, Stockholm, Sweden, December 11th, 2018 (2018/2082–31/1). Written informed consent is obtained from all parents/caregivers. The ethics committees approved the consent procedure.

### Trial status

In Sweden and Romania recruitment began in January 2019 and Spain began to recruit in February 2019. Recruitment is expected to last until 2020.

## Discussion

The ML Europe trial will assess the impact of parent support group sessions (the ML program) followed by a mHealth program (the MINISTOP app) to treat overweight and obesity in 2- to 6-year-old children from three European countries. Globally, there has been very few overweight and obesity treatment interventions targeted to pre-school aged children [6] and to date no intervention has coupled face-to-face delivery with mHealth to boost the effect of the intervention.

In this trial we aim to recruit a representative sample of the study population in each participating country. In Sweden this will be done by inviting all primary and secondary health care centers in Stockholm County to participate in recruitment, with a similar process being done in Spain (all primary health care centers and hospitals in Mallorca were invited to participate). However, the ability to get a representative sample of the study population in Romania might be more difficult as recruitment relies on families contacting the research team themselves through contacts with physicians and pediatricians and Facebook announcements. Therefore, certain parts of the population may be missed, e.g., those not likely to contact the research team and those who do not use Facebook.

Additionally, there are a few other factors that should be considered with regards to recruitment, which have the possibility to influence the representativeness of the overall sample. Firstly, the participating families need to be able to understand, speak, and read Romanian, Spanish, or Swedish sufficiently well (depending on the country of participation) in order to participate. Secondly, families with low socioeconomic status and parents with a lower educational background have been found to be less likely to participate in research [58, 59]. The inability to speak the language the intervention is being conducted in coupled with the possibility of low participation rates in families with low socioeconomic status is of concern. This is due to the fact that children of migrant parents and those of low socioeconomic status are more likely to have overweight or obesity [60, 61]. Furthermore, families will only be included if they own a smartphone compatible with the MINISTOP app, which could affect recruitment of low socioeconomic families; however, we believe this risk to be quite small as smartphones are so commonly used in most populations. Finally, we foresee that recruitment for this study will be a challenge as was found in the ML trial [7]. In the ML trial parents decided not to participate for various reasons, with the most common being parents’ work schedules or family situation [7]. When recruiting for ML Europe we used our experiences from previous clinical RCTs to ensure that recruitment and patient participation are organized in the most feasible way, e.g., time, date and place for the parent groups will be adjusted to suit as many families as possible. We anticipate the recruitment to be influenced by target population size which varies between the countries (330,000 in Timisoara, 860,000 in Mallorca and 2.3 million in Stockholm County). Also, we are aware that the prevalence of overweight and obesity among children differs in each site. While recent national data are yet to be published, in Romania, a study including 6-year-old children found the prevalence for overweight and obesity to be 19% [62]. In Spain, the prevalence of overweight and obesity was 21% in 3 to 5-year-old children [63]. In the Stockholm County, the prevalence of overweight and obesity among 4-year-old children is on average 11% ranging from 4% in the more affluent areas to over 15% in less affluent areas [64]. Thus, although the prevalence and obesity seems to be lowest in the Swedish site the larger population may compensate this challenge. It remains to be elucidated what the largest barrier in the recruitment process will be: not sufficiently large targeted population or low prevalence of overweight and obesity.

The randomized controlled design and multi-site recruitment (i.e., Timisoara, Romania; Mallorca, Spain; and Stockholm, Sweden) are strengths of this study. Furthermore, the fairly large sample size (*n* = 300) will allow us to assess the intervention’s effectiveness in samples within and across three very different European countries. With regards to the intervention, both components are based on behavior change theories (i.e., Bandura’s Social Learning Theory [40] and Patterson’s Social Interaction Learning Theory [41, 42] for the ML program and Social Cognitive Theory [65] for the MINISTOP program). Furthermore, the combination of group sessions followed by a previously evaluated mHealth app is a further strength, as it will allow for the reiteration of the material taught during the group sessions to be explained in different ways with different examples. This is important as the booster group in the ML study had a mean change in BMI z-score from baseline which was significantly larger in comparison to standard treatment and the group without boosters (− 0.54, *p* < 0.001; − 0.11, *p* = 0.551; and − 0.04 for the booster, without boosters, and standard treatment groups, respectively) [7]. In today’s society, telephone based booster sessions after an intervention such as ML is difficult to sustain due to parents’ busy schedules. Therefore, a mHealth solution such as MINISTOP to booster the effect of treatment may be a more feasible approach as it allows parents to work through the material at their own pace, when they have time. Finally, this study is limited by the fact that there is no standard overweight and obesity treatment across Europe. Therefore, the control group will receive different treatment depending on the country of participation, which could influence the results. However, standard treatment as per country is the best possible control as it would be considered unethical to withhold treatment for a condition if a treatment exists [66].

The use of objective assessments for anthropometrics and body composition, physical activity and sedentary behavior, food intake, as well as epigenetic and metabolic markers is a further strength of this study. Additionally, the use of qualitative methods, i.e., semi-structured interviews with health care professionals and parents from all sites will allow us to assess the feasibility of this new overweight and obesity management intervention in three European countries.

In conclusion, in the majority of countries, there is no standard management of overweight and obesity in the pre-school years. As overweight and obesity in this age may track into adolescence and adulthood, causing psychological and physical consequences, families should receive support as early as possible. Feasible and effective approaches for families with pre-school aged children are yet to be developed. If the ML Europe intervention is found to be effective, it has the potential to be implemented into routine care for overweight and obesity across Europe.

## Data Availability

Not applicable.

## Abbreviations

^1^H-NMR: proton nuclear magnetic resonance
App: Application
BMI: Body mass index
CEBQ: Child eating behaviour questionnaire
CFPQ: Comprehensive feeding practices questionnaire
DNA: Deoxyribonucleic acid
GIT: Gastrointestinal tract
GLP: Glucagon-like peptide
HIF3A: Hypoxia-inducible transcription factor 3A
KEEP: Keeping foster and kin parents supported and trained
MCCV-PLS-DA: Monte Carlo cross-validated partial least square-discriminant analysis
mHealth: Mobile health
MINISTOP: Mobile-based intervention intended to stop obesity in preschoolers
miR: Micro Ribonucleic acid
ML: More and Less
ncRNA: Non-coding Ribonucleic acid
PCA: Principal component analysis
PCR: Polymerase chain reaction
PLS-DA: Partial least square-discriminant analysis
PYY: Peptide YY
RCT: Randomized controlled trial
RIA: Radioimmunoassay
ROC curves: receiver operating characteristic curves
